# Supplementary figures and images for: Wrack enhancement of post-hurricane vegetation and geomorphological recovery in a coastal dune
Source: PLoS One. 2022 Aug 31;17(8):e0273258. doi: 10.1371/journal.pone.0273258 (PMC9432683; doi:10.1371/journal.pone.0273258)

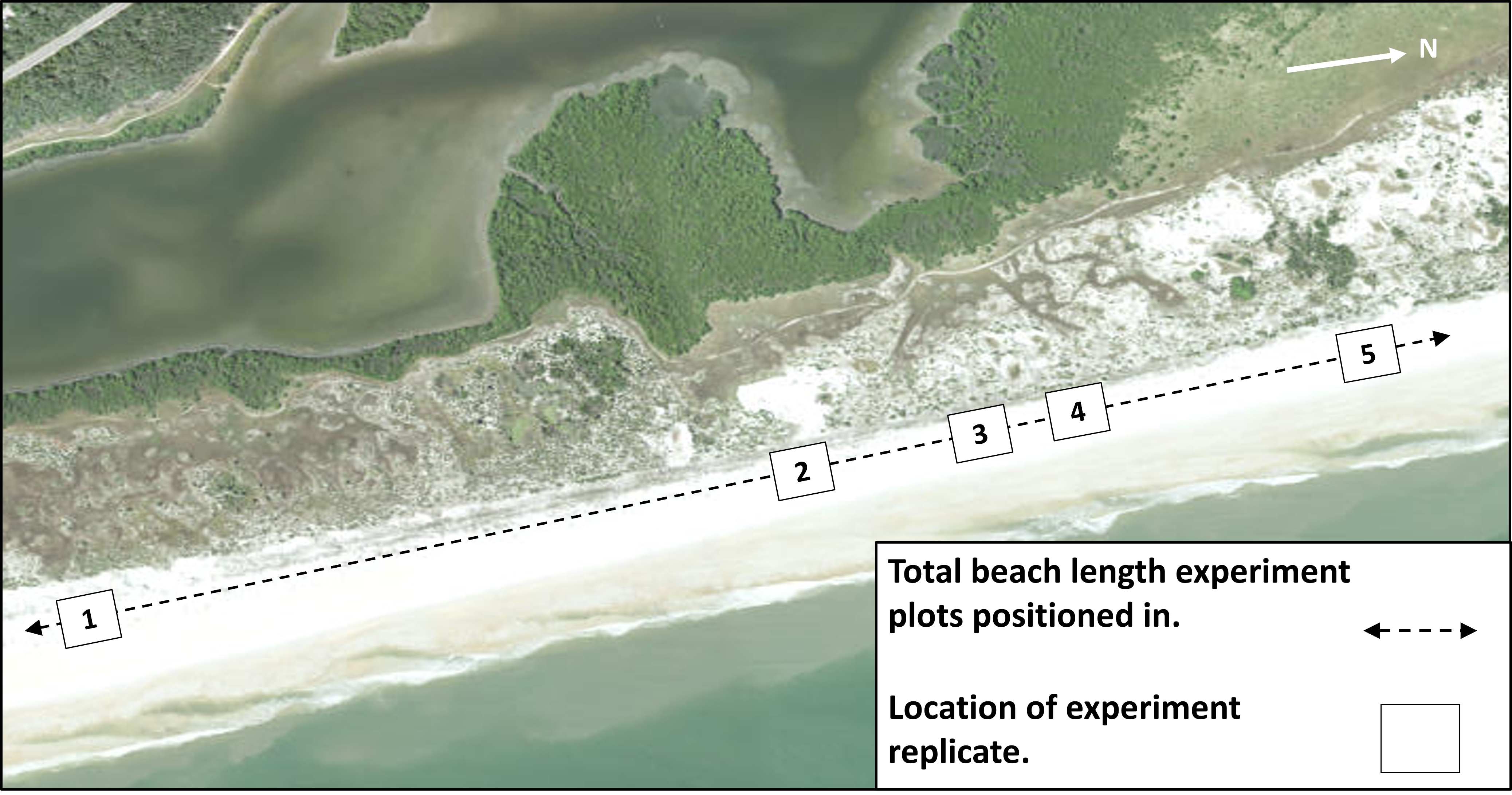

Supplement: S1 Fig — (TIF) [file pone.0273258.s001.tif]

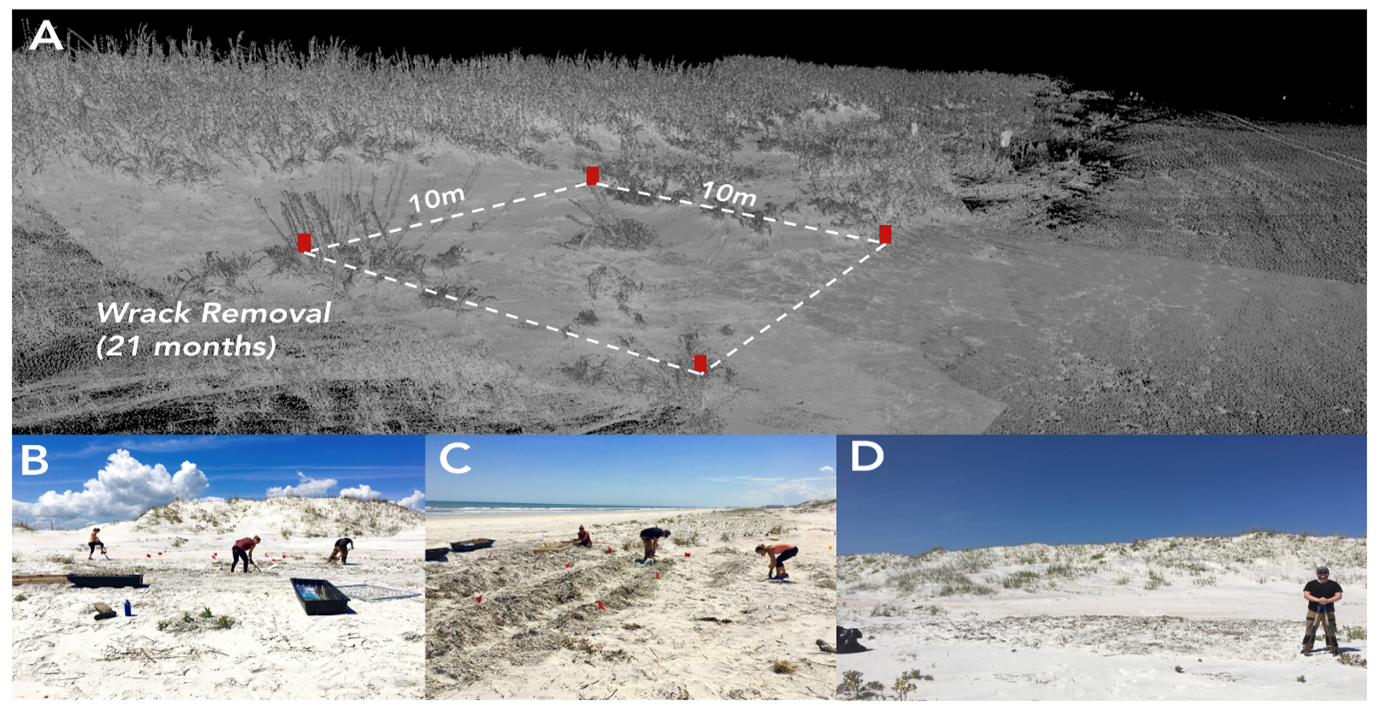

Supplement: S2 Fig — All images belong to the authors. A) Laser scan image shows removal plot 21 months after experimental deployment, red blocks represent corners of 10 x 10 m plot. B–C) Photographs of surveyors digging wrack out of removal plot prior to sieving process during experiment deployment. D) Photograph showing removal plot (bare area of sand to rear left of surveyor) immediately after deployment completed. (TIFF) [file pone.0273258.s002.tiff]

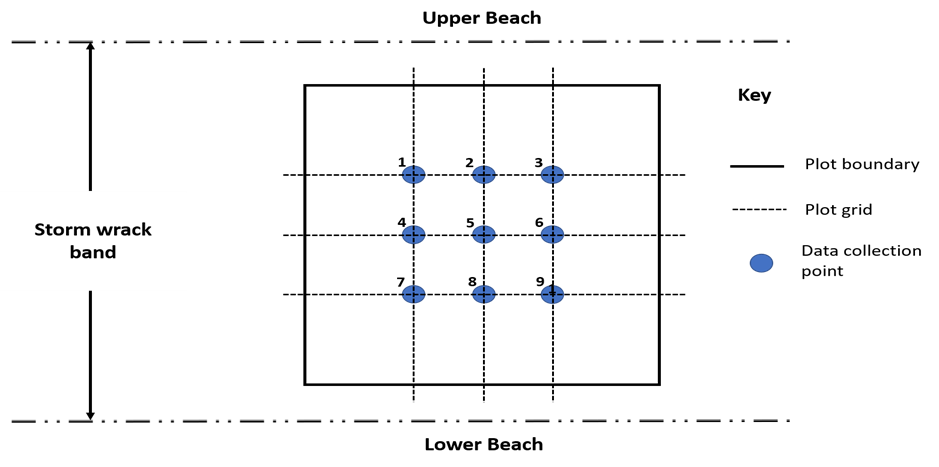

Supplement: S3 Fig — Schematic shows nine data collection points at bisecting points of lateral orthogonal transects three, five and seven metres along on plot axes. Data collection points show positions where 0.25 m2 quadrats were used to estimate vegetation abundance. (TIFF) [file pone.0273258.s003.tiff]

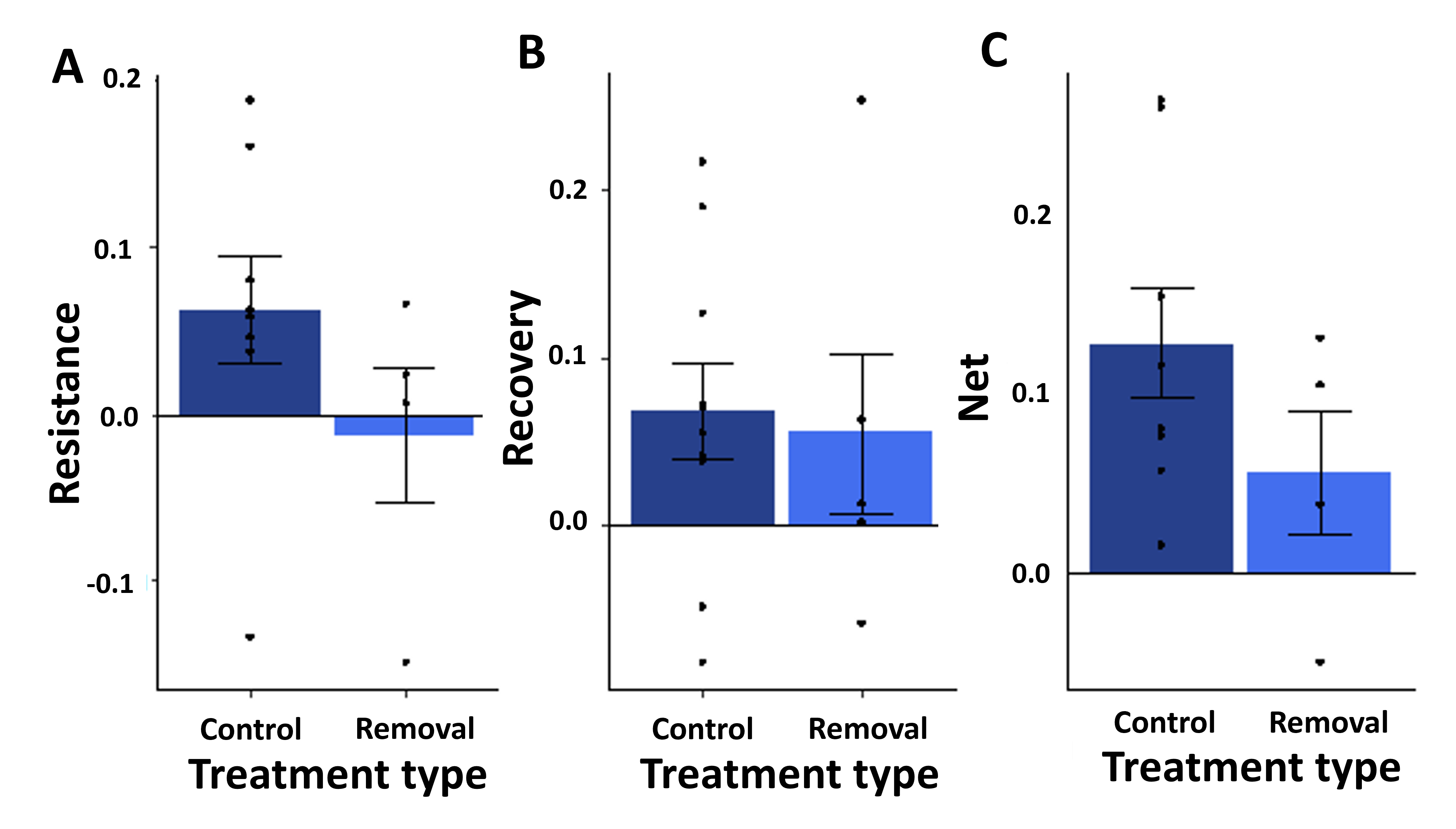

Supplement: S4 Fig — All plots show treatment type (control vs removal) and standard error. (TIF) [file pone.0273258.s004.tif]

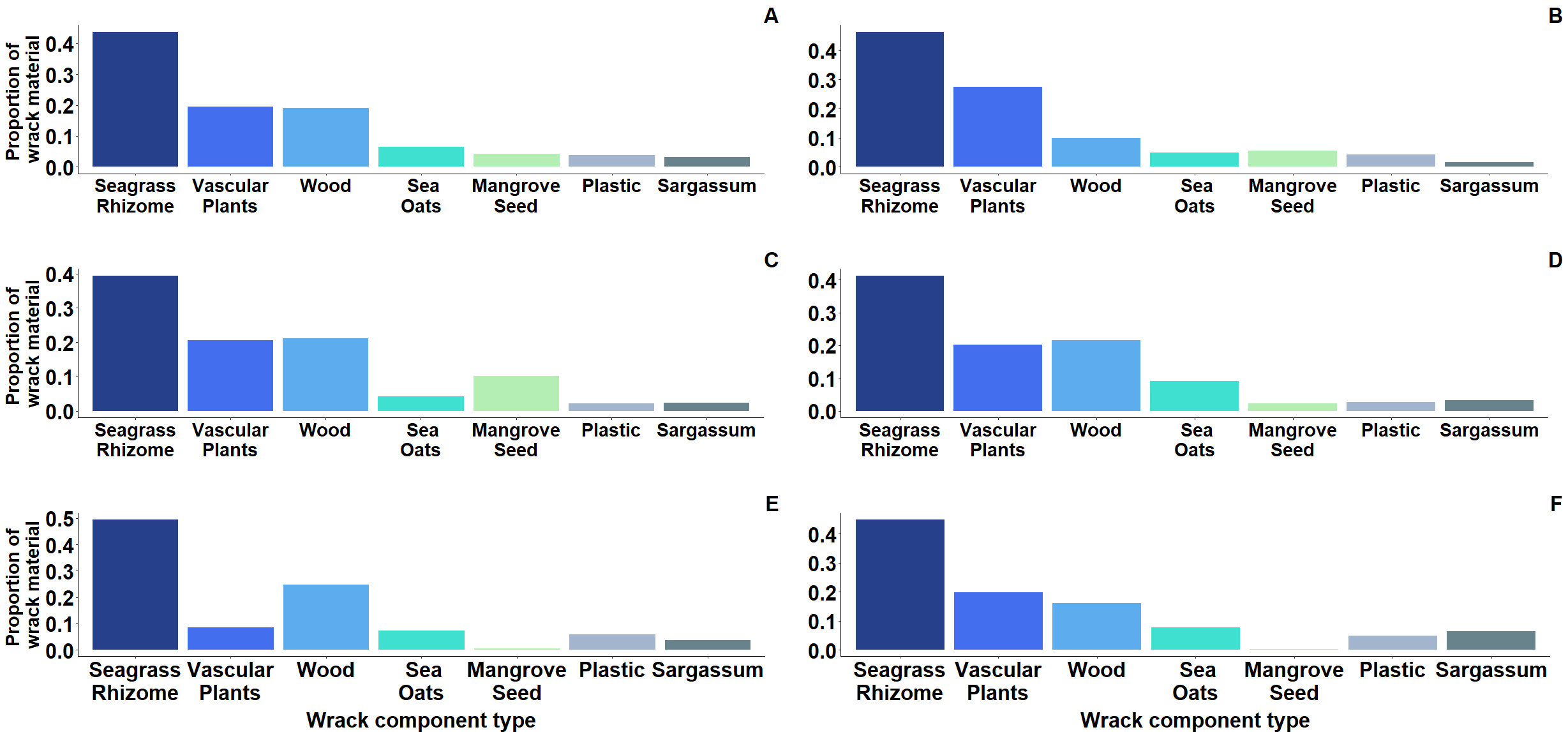

Supplement: S5 Fig — Fig shows the proportions of different components of storm wrack removed from all blocks combined (A) and from individual experimental plots (B-F). (TIF) [file pone.0273258.s005.tif]

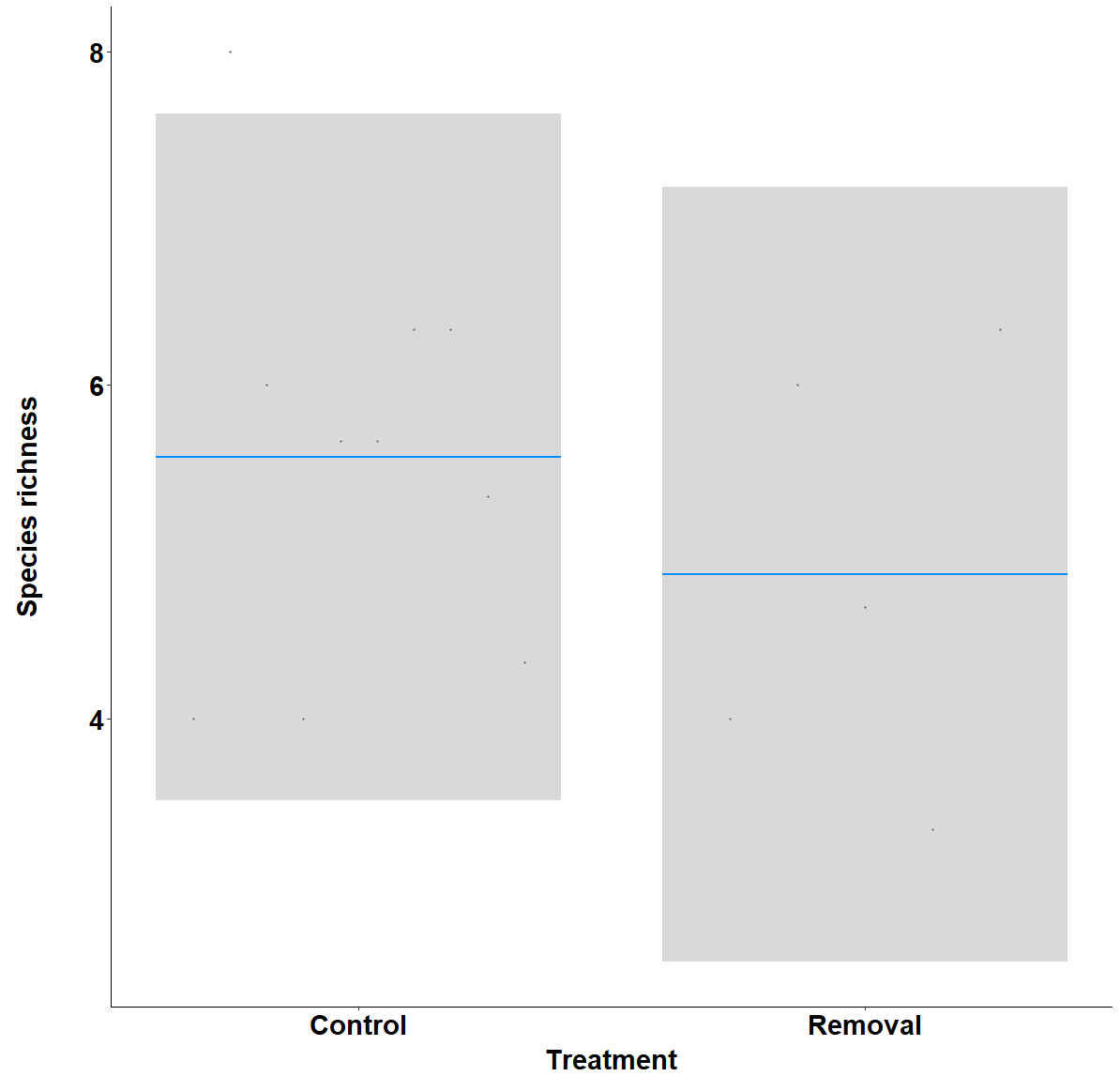

Supplement: S6 Fig — Plot shows the partial residuals of linear model showing the predicted species richness as a function of treatment type (control vs removal). Measurements taken after 15 months. No significant difference detected (t = -0.850, p = 0.42). Grey area represents the 95% confidence intervals of the means. (TIF) [file pone.0273258.s006.tif]

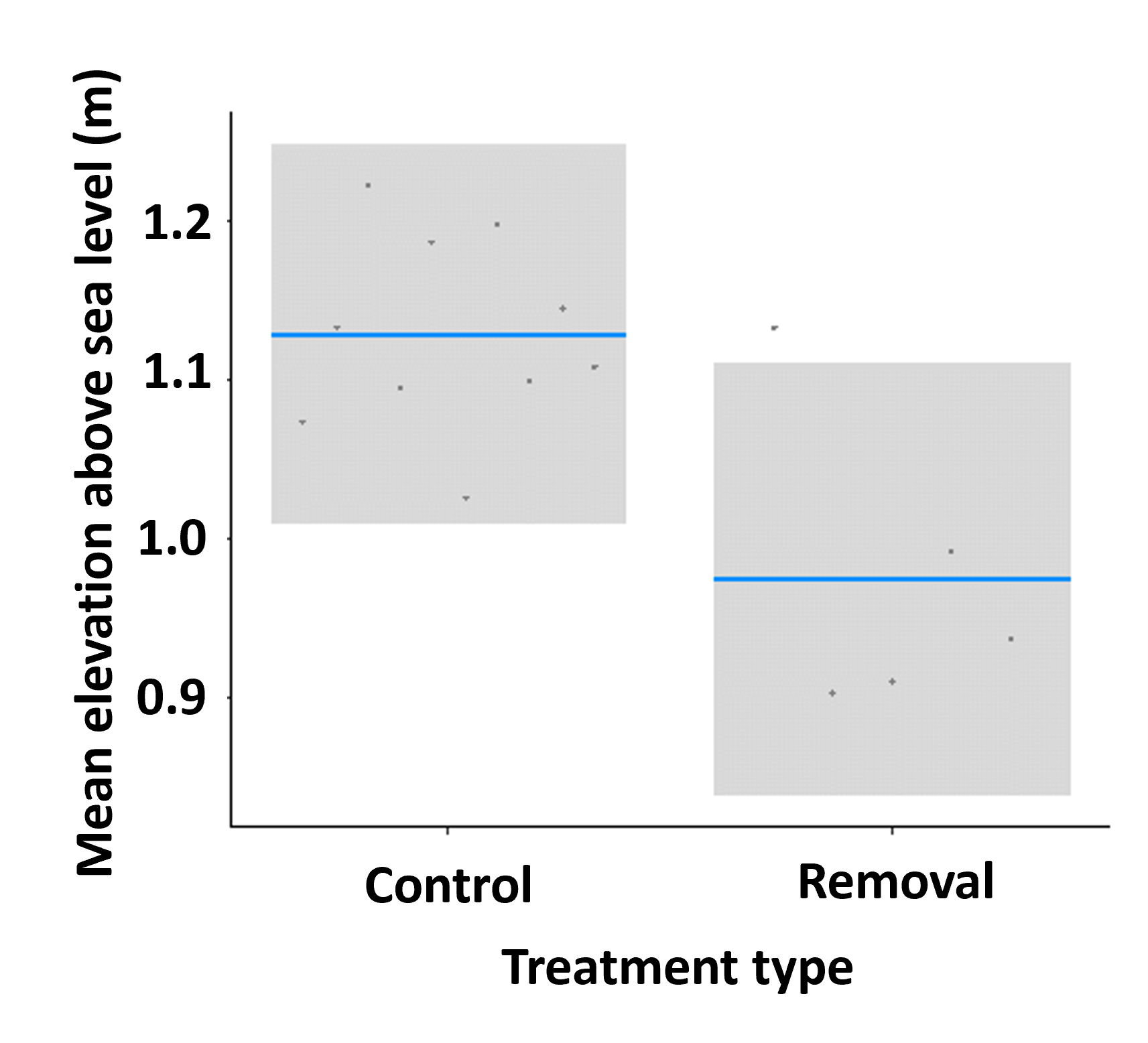

Supplement: S7 Fig — Plot shows the partial residuals from linear models accounting for experimental block as a function of treatment type (control vs removal). Significant effect detected (t = -3.19, p = 0.011) Elevation response derived from the average of three data collection time points (August, September and December 2019). Grey area represents the 95% confidence of the means. (TIF) [file pone.0273258.s007.tif]
