# Supplementary material for: Wrack enhancement of post-hurricane vegetation and geomorphological recovery in a coastal dune
Source: PLoS One. 2022 Aug 31;17(8):e0273258. doi: 10.1371/journal.pone.0273258 (PMC9432683; doi:10.1371/journal.pone.0273258)
Supplement: S1 Table — (DOCX) [file pone.0273258.s008.docx]

| Site | Latitude | Longitude |
| --- | --- | --- |
| Cape Canaveral Site 1 | 28˚53’5.94”N | -80˚47’37.19”W |
| Cape Canaveral Site 2 | 28˚54’39.57”N | -80˚48’40.52”W |
| East 7th Street | 29˚1’34.89”N | -80˚53’18.16”W |
| Crawford Road | 29˚2’38.00”N | -80˚53’54.12”W |
| Ocean Drive | 29˚3’36.48”N | -80˚54’21.89”W |
| Amsden Road | 29˚18’36.53”N | -81˚2’51.98”W |
| Daytona Beach | 29˚13’3.28”N | -81˚0’8.42”W |
| Flagler Beach | 29˚25’37.67”N | -81˚6’8.50”W |
| Beverley RV Park | 29˚31’22.16”N | -81˚8’51.78”W |
| Mala Compra Road | 29˚36’57.98”N | -81˚11’24.73”W |
| Marineland | 29˚40’15.97”N | -81˚12’49.07”W |
| Mantanzas Bay | 29˚43’2.64”N | -81˚13’51.53”W |
| Butler Beach | 29˚47’11.30”N | -81˚15’25.57”W |
| Crescent Beach | 29˚45’44.69”N | -81˚14’58.52”W |
| St Augustine Beach | 29˚51’22.49”N | -81˚15’53.49”W |
| Surfside Park | 29˚55’25.08”N | -81˚17’35.87”W |
| South Vilano Beach | 29˚56’24.6”N | -81˚18’1.12” |
| North Vilano Beach | 30˚6’56.33”N | -81˚20’40.55W |
| Ponte Vedra Beach | 30˚14’32.08”N | -81˚22’39.87”W |
| Atlantic Road | 30˚20’47.58”N | -81˚23’47.85”W |
| Fernandina Beach | 30˚38’37.66”N | -81˚26'4.83"W |
